# Pre-operative clonal hematopoiesis is related to adverse outcome in lung cancer after adjuvant therapy

**DOI:** 10.1186/s13073-023-01266-4

**Published:** 2023-12-12

**Authors:** Jae Kwang Yun, Sugyeong Kim, Hongyul An, Geun Dong Lee, Hyeong Ryul Kim, Yong-Hee Kim, Dong Kwan Kim, Seung-Il Park, Sehoon Choi, Youngil Koh

**Affiliations:** 1 Department of Thoracic and Cardiovascular Surgery, Asan Medical Center, Ulsan University College of Medicine, 88, Olympic-Ro 43-Gil, Songpa-Gu, Seoul, Republic of Korea; 2 Genome Opinion Inc., Sungsu SKV1 Center, 1-721, 48, Achasan-Ro 17-Gil, Seongdong-Gu, Seoul, Republic of Korea; 3 Department of Internal Medicine, Seoul National University College of Medicine, Seoul National University Hospital, 101, Daehak-Ro, Jongno-Gu, Seoul, Republic of Korea

**Keywords:** Clonal hematopoiesis, Non–small cell lung cancer, Adjuvant therapy, Prognosis

## Abstract

**Background:**

Clonal hematopoiesis (CH) frequently progresses after chemotherapy or radiotherapy. We evaluated the clinical impact of preoperative CH on the survival outcomes of patients with non-small cell lung cancer (NSCLC) who underwent surgical resection followed by adjuvant therapy.

**Methods:**

A total of 415 consecutive patients with NSCLC who underwent surgery followed by adjuvant therapy from 2011 to 2017 were analyzed. CH status was evaluated using targeted deep sequencing of blood samples collected before surgery. To minimize the possible selection bias between the two groups according to CH status, a propensity score matching (PSM) was adopted. Early-stage patients were further analyzed with additional matched cohort of patients who did not receive adjuvant therapy.

**Results:**

CH was detected in 21% (86/415) of patients with NSCLC before adjuvant therapy. Patients with CH mutations had worse overall survival (OS) than those without (hazard ratio [95% confidence interval] = 1.56 [1.07–2.28], *p* = 0.020), which remained significant after the multivariable analysis (1.58 [1.08–2.32], *p* = 0.019). Of note, the presence of CH was associated with non–cancer mortality (*p* = 0.042) and mortality of unknown origin (*p* = 0.018). In patients with stage IIB NSCLC, there was a significant interaction on OS between CH and adjuvant therapy after the adjustment with several cofactors through the multivariable analysis (HR 1.19, 95% CI 1.00–1.1.41, *p* = 0.041).

**Conclusions:**

In resected NSCLC, existence of preoperative CH might amplify CH-related adverse outcomes through adjuvant treatments, resulting in poor survival results.

**Supplementary Information:**

The online version contains supplementary material available at 10.1186/s13073-023-01266-4.

## Background

Clonal hematopoiesis (CH) is a condition defined by the expansion of clonally derived hematopoietic stem cells (HSCs) that harbor somatic mutations in leukemia-associated genes, which can be detected by next-generation sequencing (NGS) [1–3]. CH is associated with aging [4, 5] and has a significant association with prior radiation therapy (RTx) and/or prior exposure to chemotherapy (CTx) [6]. CH is contributing to the development of cardiovascular diseases and hematological malignancies [1, 4, 7, 8]. Mechanistically, CH causes cardiovascular disease as a result of mutated genes altering the inflammatory response, a well-known contributing factor for developing atherosclerosis [3]. Indeed, the clinical significance of CH in various chronic disease, infectious disease, and malignancy has recently started to gain attraction [6, 9–11].

Of particular interest is the impact of CH on cancer survivors who have previously undergone cancer-related therapy [6, 11]. Considering the altered immune response by CH, CH may also alter the clinical consequences of the cancer [6]. In addition, CH may also play an important role in the morbidity such as cardiovascular disease in cancer survivors [12]. Hence, an in-depth study into how CH might influence cancer recurrence and response to therapy will help to decide the surveillance protocol, such as screening, follow-up duration, and risk-directed therapeutic approaches for high-priority groups.

Lung cancer is the most commonly diagnosed cancer and the leading cause of cancer-related deaths worldwide [13]. Recent studies have demonstrated that CH is common in patients with solid tumors including lung cancer [6, 11]. Given the critical role of inflammation in the pathogenesis of lung cancer [14], there is a possibility that CH influences the prognosis in patients with non-small cell lung cancer (NSCLC). Although several prognostic factors, such as age, sex, and cancer stage, have been identified [15], further study to determine novel factors in the era of NGS is highly encouraged. Of note, as cytotoxic chemotherapy and radiotherapy significantly contribute to the development and progression of CH, there is a chance for the existence of interaction between CH status and oncologic benefit of adjuvant therapy in resected NSCLC.

In this study, we evaluated the clinical impact of preoperatively existing CH on the cancer recurrence and survival in patients with NSCLC who received surgical resection followed by adjuvant therapy using a large-scale single center consecutive surgical cohort [16]. We assumed that preoperative existence of CH may adversely affect survival outcome in NSCLC via non-cancer mortality when they are exaggerated with adjuvant treatment.

## Methods

### Patients

**Fig. 1:**
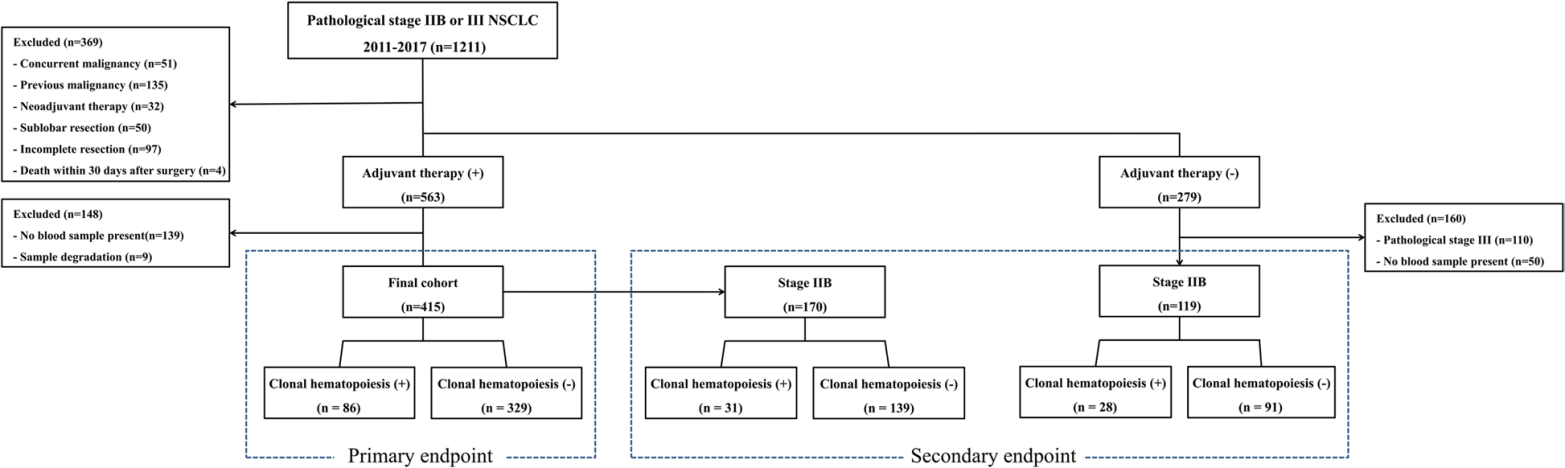
CONSORT diagram

All clinical records of patients who underwent surgery for NSCLC between January 2011 and December 2017 were reviewed from the lung cancer database of Asan Medical Center, Seoul, Korea. The study was conducted in patients with pathological stage IIB or III NSCLC for which adjuvant therapy was indicated (Fig. 1). The exclusion criteria were as follows: (i) patients with previous or current malignancy other than lung cancer, (ii) patients who received neoadjuvant therapy, (iii) patients who underwent sublobar resection (wedge resection or segmentectomy), (iv) patients with incomplete resection, and (v) patients who died within 30 days after surgery. After exclusion according to the criteria, we identified 563 patients who underwent adjuvant CTx or chemoradiation therapy (CRTx) for pathological stage IIB or III NSCLC. Among them, there were 424 patients with blood samples that were collected before surgery and stored in Asan Bio-Resource Center, Korea Biobank Network. Of these, 9 samples were excluded due to sample degradation, and 415 patients with blood samples were enrolled as final cohort.

Additional analysis was performed to identify the clinical impact of CH according to the adjuvant therapy in patients with early-stage NSCLC where oncologic benefit of adjuvant therapy is marginal. Accordingly, blood samples of NSCLC patients with stage IIB who did not perform adjuvant therapy for stage IIB NSCLC were additionally analyzed (Fig. 1).

### Sample processing and sequencing

CH blood-derived DNA from a patient was used for targeted NGS with a custom panel containing 89 genes which were selected from previously reported literature used as CH variants. The sequencing libraries were prepared following the SureSelect XT HS Target Enrichment System (Agilent, Santa Clara, CA) protocol. The libraries were sequenced on the Illumina NovaSeq6000 platform (Illumina, San Diego, CA) with 150 bp paired-end following the manufacturer’s protocols. The mean depth of coverage of an analysis ready BAM was more than 800 × . Sequencing reads were processed using software tools like Illumina’s bcl2fastq (v2.17.1.14), SeqPrep, Sickle (v1.33), and GATK, which help in trimming, alignment, and marking duplicates, resulting in an analysis-ready BAM file for each sample. To identify and eliminate artifactual variants, two CHIP negative cohorts were used based on different enrichment reagents. For variant calling, BAM files were examined using a trio of somatic variant calling tools, including VarDict, Mutect2(4.1.4.1), and SNVer(0.4.1). Final variants were identified by applying various filters, including read counts, variant allele frequency (VAF), and comparison against established databases like gnomAD and COSMIC, followed by a detailed review in IGV to exclude potential artifacts.

### Postoperative management

Adjuvant CTx was recommended for all patients with stage II or more, except when the patient was above 75 years of age or in poor physical condition, according to the judgment of the multidisciplinary team. Systemic CTx with a platinum-based regimen was recommended for 4–6 weeks after surgery, with a total of four cycles of treatment. A tyrosine kinase inhibitor was used for patients with activating mutations in the epidermal growth factor receptor (EGFR) mainly when recurrence occurred after the first adjuvant CTx. For adjuvant RTx, a daily dose of 1.8 Gy was administered up to a total dose of 50.4 Gy for patients with pathological N2 disease who underwent complete resection or 55–60 Gy for patients with positive resection margins. Among patients who underwent complete resection, a considerable number of patients with single N2 node metastasis skipped adjuvant RTx.

### Definitions

The primary end point was survival outcomes following the presence of CH in patients who underwent adjuvant therapy for stage IIB or III NSCLC. Secondary end points included survival outcomes of patients with stage IIB according to the presence of CH and the performance of adjuvant therapy (Fig. 1).

Eighty-nine genes frequently detected in CH were custom selected, and a variant allele frequency (VAF) of ≥ 2.0% was set as the cut-off for carrier, which has been adopted in previous studies (Additional file 1: Table S1) [1, 2, 4, 7, 17, 18].

Overall survival (OS) was defined as the time interval between the date of operation and the date of death, which was determined by reviewing the records from the Korean National Security Death Index Database. Lung cancer mortality included deaths resulting from evident tumor progression. Non-lung cancer mortality was defined as deaths with a known cause not due to lung cancer progression. Recurrence-free survival (RFS) was calculated as the time between the date of resection and the date of recurrence, and patients without recurrence were censored at the latest timepoint known to be recurrence-free.

### Statistical analysis

Continuous variables are presented as means and standard deviations, and categorical variables as count and percentage. Student’s *t*-test or Wilcoxon rank–sum test was used to compare the two groups in terms of continuous variables, and the chi-square test or Fisher’s exact test was applied for categorical variables.

The OS and RFS outcomes were defined using Kaplan–Meier curves. The differences in the survival rates were analyzed using the log-rank test. A Cox proportional hazards model was used for the univariable and multivariable analyses to identify the clinical impact of CH on survival outcomes. After exclusion of the correlated variables, independent variables with *p* ≤ 0.05 in univariate analysis were entered into the initial multivariate Cox model. The final multivariable model was selected using the forward stepwise selection (*p* ≤ 0.10 for entering the model and *p* ≤ 0.05 for staying in the model). The proportional hazards assumption for the Cox regression models was tested using Schoenfeld residuals.

Considering the correlative effect of CH with other covariates, such as age, sex, and smoking history, a propensity score matching (PSM) technique was used to exclude possible selection bias according to these variables, which could not be adjusted in the final multivariable Cox model. After PSM, the McNemar’s test and paired *t*-tests were used to analyze the propensity score-matched pairs. As the two cause-specific deaths are mutually exclusive, significant differences in cumulative incidence function values among subgroups were evaluated using Gray’s test [19]. For PSM, observation pairs with equivalent propensity scores were selected with nearest-neighbor matching and a caliper width of 0.25 of the standard deviation. CH-negative patients were randomly matched to CH-positive patients at a ratio of 2:1. Balance between the groups was assessed using standardized mean differences (SMDs). An absolute standardized difference of ≤ 0.1 was considered to indicate the ideal balance and that of ≤ 0.2 was considered to indicate acceptable balance [20].

All statistical calculations were performed using R version 4.0.2 (The R Foundation for Statistical Computing, Vienna, Austria) using the “Survival,” “MatchIt,” “cmprsk,” “dplyr,” “sad,” “ggplot2,” “GGally,” “survminer,” and “rms” packages. All reported *p*-values are two-sided. *p*-values < 0.05 were considered significant.

## Results

### Characteristics of CH

The mean age of the patients in the cohort was 60.2 ± 8.3 years. Of the total 415 patients, CH was found in 86 (20.7%) patients. The prevalence of CH was 10.4%, 14.9%, 23.8%, and 34.5% in patients in their 40s, 50s, 60s, and 70s, respectively, showing a continuous increase with age (Additional file 1: Fig S1). As for the number of mutations, single mutation was the most common in 82.6% of patients, two mutations in 14.0%, and three mutations in 3.5% (Additional file 1: Fig S1). Mutations in DNMT3A (33.0%) were the most common, followed by ASXL1 (13.2%), TET2 (11.3%), and PPM1D (7.5%); these four genes accounted for 65.1% of all mutations detected (Additional file 1: Fig S1). Details of detected CH mutations for individual patients are summarized in the Additional file 1: Table S2.

### Patient characteristics

The mean postoperative follow-up duration was 44.6 ± 24.3 months. The baseline demographics of the patients and tumor characteristics are listed in Table 1. Before PSM, patients with CH (*n* = 86) were older (*p* < 0.001) than those without (*n* = 329). There were no significant differences in sex (*p* = 0.367), smoking history (*p* = 0.785), the number of comorbidities (*p* = 0.988), the rate of EGFR mutation (*p* = 0.501), the distribution of histology (*p* = 0.647) and overall stage (*p* = 0.548), and the type of adjuvant therapy (*p* = 0.146) between the two groups. After PSM, all variables, including age (*p* = 0.620), became similar between the two groups and were well balanced (all SMDs < 0.2) (Additional file 1: Table S3.).

**Table 1:** Baseline characteristics of patients who underwent adjuvant therapy for stage IIB or III according to the presence of clonal hematopoiesis (final cohort)

Variables	Total (*n* = 415)	CH ( +) (*n* = 86)	CH ( −) (*n* = 329)	*p*-value
**Age (year)**	60.2 ± 8.3	63.0 ± 8.2	59.5 ± 8.2	0.001*
**Sex (male)**	270 (65.1)	60 (69.8)	210 (63.8)	0.367
**History of smoking**	248 (59.8)	53 (61.6)	195 (59.3)	0.785
**The number of comorbidities**				0.988
0	225 (54.2)	46 (53.5)	179 (54.4)	
1	123 (29.6)	26 (30.2)	97 (29.5)	
≥ 2	67 (16.1)	14 (16.3)	53 (16.1)	
**Pulmonary function**				
FEV1 < 60%	17 (4.1)	4 (4.7)	13 (4.0)	1.000
DLCO < 60%	25 (6.0)	6 (7.0)	19 (5.8)	0.871
**Histologic structure**				0.647
ADC*		54 (62.8)	220 (66.9)	
SqCC*		24 (27.9)	87 (26.4)	
Others		8 (9.3)	22 (6.7)	
**Maximal tumor size (mm)**	40.6 ± 17.6	40.6 ± 17.3	40.6 ± 17.7	0.997
**EGFR mutation**				0.501
Yes	110 (26.5)	22 (25.6)	88 (26.7)	
No	124 (29.9)	22 (25.6)	102 (31.0)	
Unchecked	181 (43.6)	42 (48.8)	139 (42.2)	
**Pathological T factor**				0.280
T1	81 (19.5)	22 (25.6)	59 (17.9)	
T2	186 (44.8)	32 (37.2)	154 (46.8)	
T3	110 (26.5)	25 (29.1)	85 (25.8)	
T4	38 (9.2)	7 (8.1)	31 (9.4)	
**Pathological N factor**				0.791
N0	43 (10.4)	9 (10.5)	34 (10.3)	
N1	182 (43.9)	35 (40.7)	147 (44.7)	
N2	190 (45.8)	42 (48.8)	148 (45.0)	
**Pathological stage**				0.548
IIB	170 (41.0)	31 (36.0)	139 (42.2)	
IIIA	192 (46.3)	44 (51.2)	148 (45.0)	
IIIB	53 (12.8)	11 (12.8)	42 (12.8)	
**Type of adjuvant therapy**				0.146
CTx	248 (59.8)	45 (52.3)	203 (61.7)	
CRTx	167 (40.2)	41 (47.7)	126 (38.3)	

Data are presented as no. (%) unless noted otherwise. Student’s t-test was used to compare the two groups in terms of continuous variables, and the chi-square test or Fisher’s exact test was applied for categorical variables

*CH* Clonal hematopoiesis, *FEV1* Forced expiratory volume during the first second, *DLCO* Diffusing capacity for carbon monoxide, *ADC* Adenocarcinoma, *SqCC* Squamous cell carcinoma, *EGFR* Epidermal growth factor receptor, *CRTx* Chemoradiotherapy, *CTx* Chemotherapy

^*^ denotes *p* < 0.05

### Survival analysis

Overall, 45 patients with CH (*n* = 86) and 124 patients without CH (*n* = 329) had died by the end of follow-up, and their 5-year OS rates were 45.1% and 61.9%, respectively. Recurrence events occurred in 48 and 161 patients with and without CH, respectively, and their 5-year RFS rates were 39.1% and 44.2%, respectively. Detailed information for the cause of death is summarized in Additional file 1: Table S4.

**Fig. 2:**
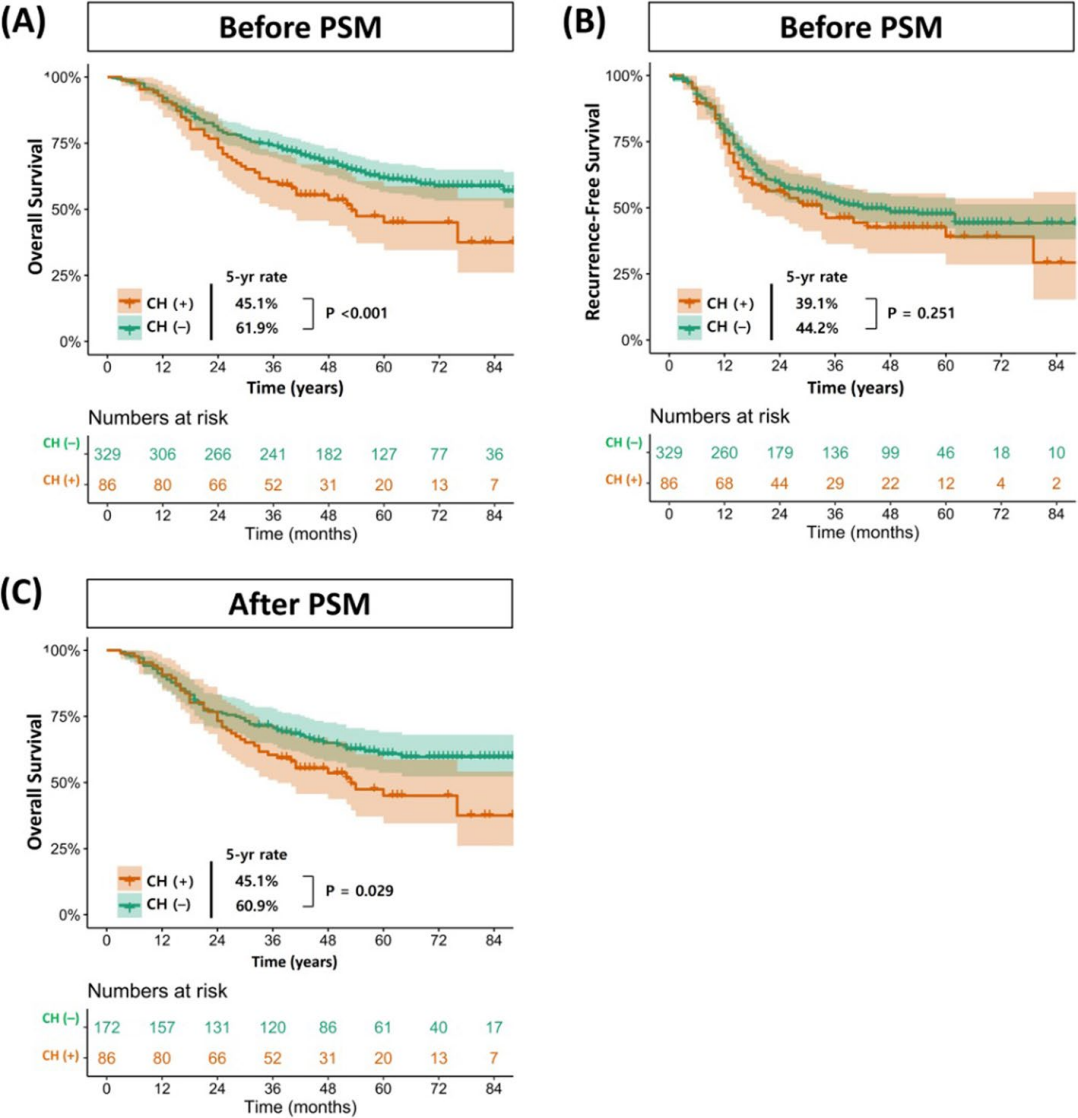
Overall survival (**A**) and recurrence-free survival (**B**) of patients following the presence of CH mutations in the entire cohort. Overall survival (**C**) of patients following the presence of CH mutations after PSM. CH, clonal hematopoiesis; PSM, propensity score matching

**Fig. 3:**
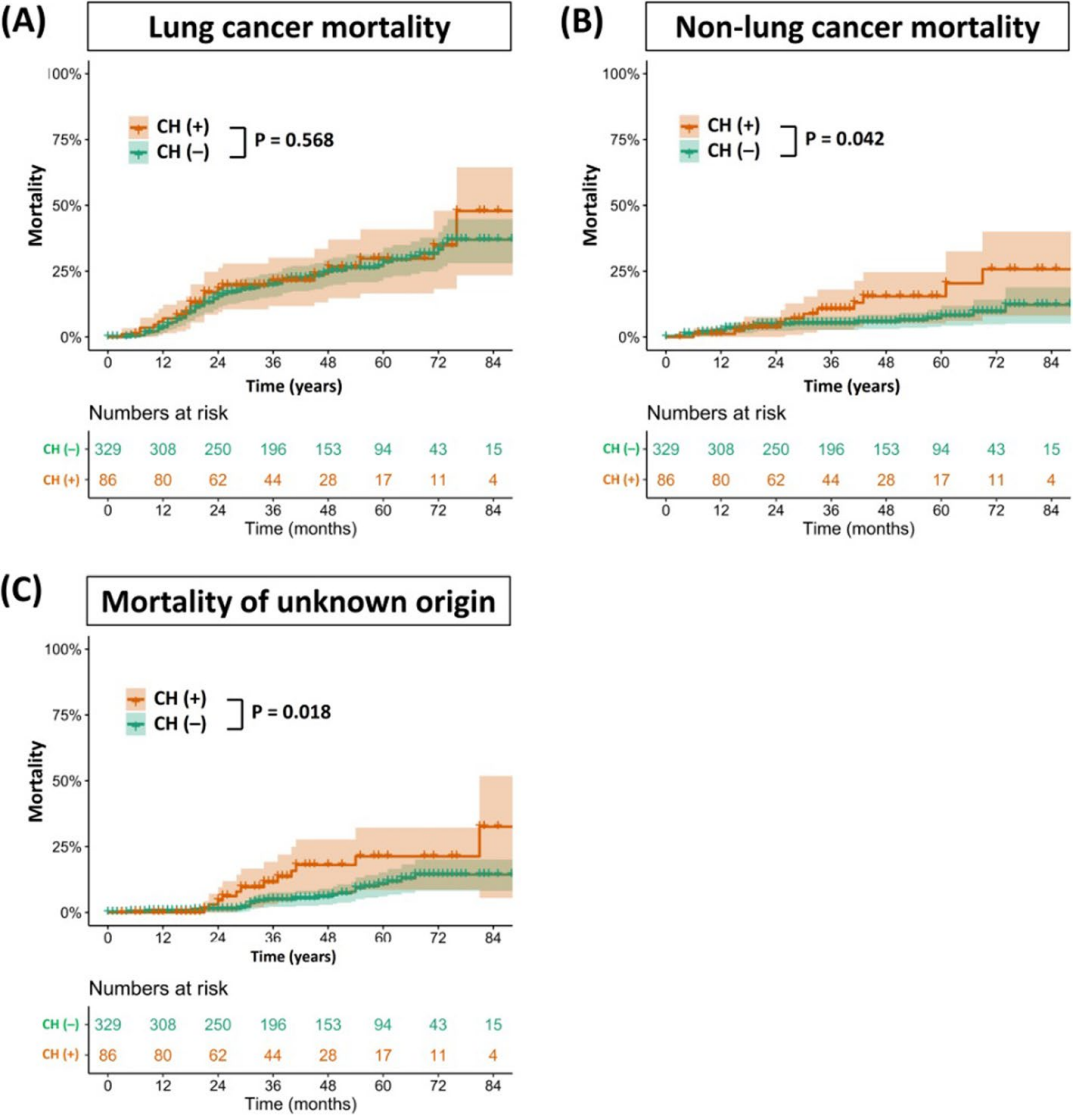
**A** Cumulative lung cancer mortality according to the presence of CH. **B** Cumulative non–lung cancer mortality according to the presence of CH. **C** Cumulative mortality of unknown origin according to the presence of CH. CH, clonal hematopoiesis

The Kaplan–Meier survival curves according to the presence of CH are plotted in Fig. 2. While there was no significant difference in RFS between the two groups (*p* = 0.251), patients with CH had worse OS than those without CH (*p* < 0.001) (Fig. 2A, B). After PSM, patients with CH still had a significantly worse survival rate than those without CH (*p* = 0.029) (Fig. 2C). According to the cause of death, lung cancer mortality was similar regardless of CH (*p* = 0.568). However, patients with CH had a statistically higher non–lung cancer mortality (*p* = 0.042) and mortality of unknown origin (*p* = 0.018) compared to those without CH (Fig. 3).

**Fig. 4:**
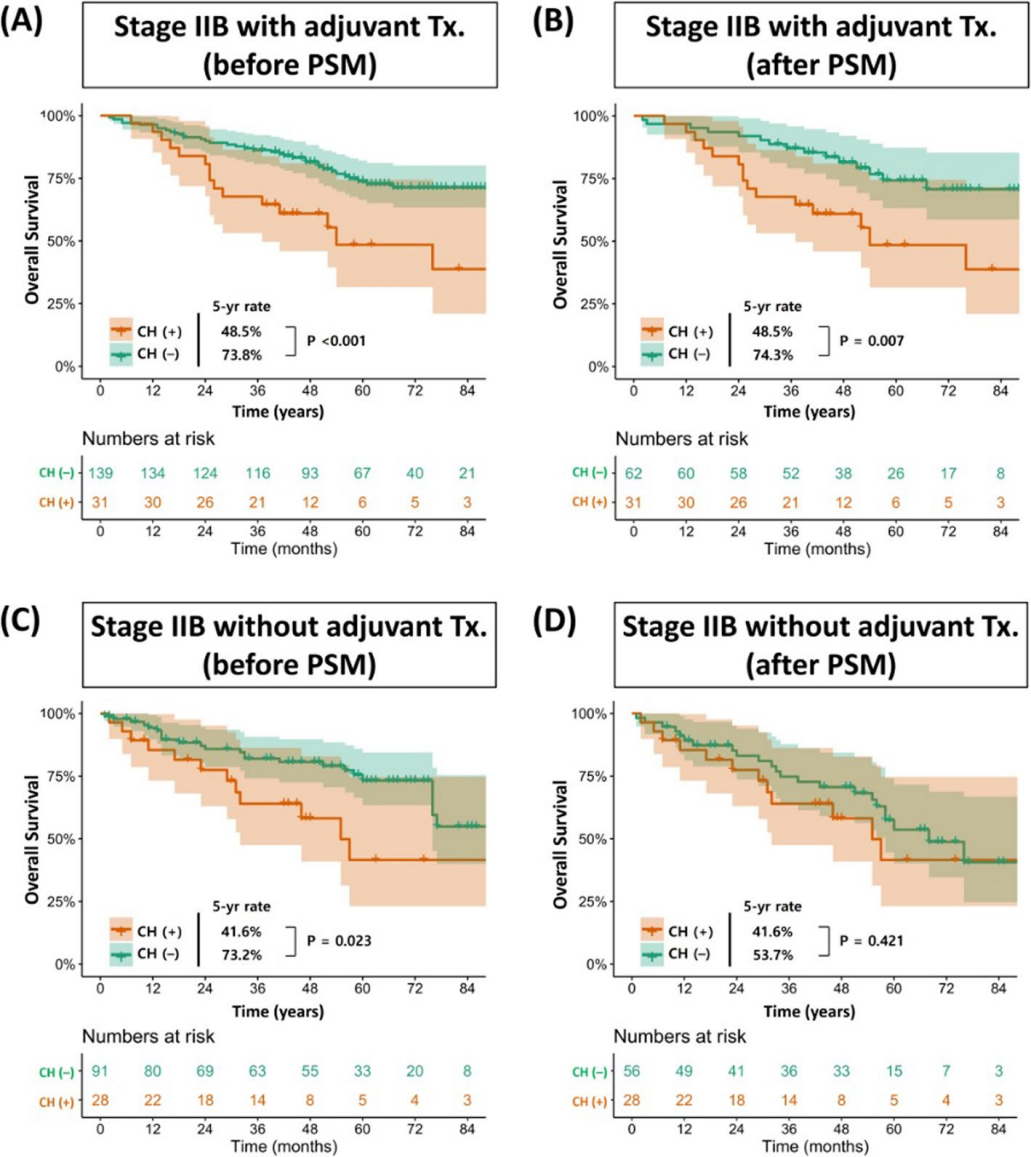
Overall survival following the presence of CH mutations in patients who underwent adjuvant therapy for stage IIB lung cancer before (**A**) and after PSM (**B**). Overall survival following the presence of CH mutations in patients who did not perform adjuvant therapy for stage IIB lung cancer before (**C**) and after PSM (**D**). CH, clonal hematopoiesis; PSM, propensity score matching; Tx, therapy

In multivariable Cox analysis, the presence of CH, along with diffusing capacity for carbon monoxide < 60%, the rate of EGFR mutation, histologic type, and tumor stage, was a significant prognostic factor for OS in patients with advanced NSCLC who underwent adjuvant therapy (hazard ratio [HR] [95% confidence interval] = 1.56 [1.07–2.28], *p* = 0.020) (Table 2) (Fig. 4). Although age was a significant factor in univariable analysis (HR [95% confidence interval] = 1.02 [1.00–1.04], *p* = 0.047), it became insignificant after the adjustment with several covariates, including the presence of CH (HR [95% confidence interval] = 1.01 [0.99–1.04], *p* = 0.173).

**Table 2:** Univariable and multivariable analysis for overall survival of all patients

	Univariable analysis		Multivariable analysis	
	HR* (95% CI)	*p*-value	HR (95% CI)	*p*-value
**Presence of CH (VAF ≥ 2.0%)**	1.56 (1.07–2.28)	0.020*	1.58 (1.08–2.32)	0.019*
**Age (years)**	1.02 (1.00–1.04)	0.047*		
**Sex (male)**	1.06 (0.77–1.46)	0.711		
**History of smoking**	1.19 (0.87–1.63)	0.265		
**The number of comorbidities**				
1 vs. 0	0.82 (0.57–1.20)	0.307	0.85 (0.58–1.25)	0.407
≥ 2 vs. 0	1.97 (1.36–2.85)	< 0.001*	1.67 (1.12–2.48)	0.011*
**Pulmonary function**				
FEV1 < 60%	1.15 (0.54–2.45)	0.722		
DLCO < 60%	1.78 (1.03–3.07)	0.040*	2.22 (1.24–3.96)	0.007*
**EGFR mutation**				
No vs. yes	2.29 (1.55–3.39)	< 0.001*	2.41 (1.59–3.65)	< 0.001*
Unchecked vs. yes	2.68 (1.83–3.90)	< 0.001*	3.11 (2.09–4.64)	< 0.001*
**Histologic structure**				
SqCC vs. ADC	0.88 (0.61–1.26)	0.482		
Others vs. ADC	1.38 (0.79–2.40)	0.258		
**Pathological stage**				
IIIA vs. IIB	1.83 (1.29–2.58)	0.001*	1.49 (1.01–2.19)	0.045*
IIIB vs. IIB	2.69 (1.72–4.21)	< 0.001*	2.18 (1.32–3.60)	0.002*
**Adjuvant therapy**				
CTx vs. CRTx	0.78 (0.57–1.05)	0.103		

Univariable and multivariable cox regression a﻿nalysis were used for comparison

*OS* Overall survival, *HR* Hazard ratio, *CI* Confidence interval, *CH* Clonal hematopoiesis, *VAF* Variant allele fraction, *ADC* Adenocarcinoma, *SqCC* Squamous cell carcinoma, *CH* Clonal hematopoiesis, *CRTx* Chemoradiotherapy, *CTx* Chemotherapy

^*^ denotes *p* < 0.05

Among the mutated genes, genes related to DNA damage response (DDR) including PPM1D, TP53, and CHEK2 was associated with worse OS in both univariable (HR [95% confidence interval] = 2.12 [1.00–4.56], *p* = 0.045) and multivariable analysis (HR [95% confidence interval] = 2.32 [1.08–4.98], *p* = 0.031) (Additional file 1: Table S5). Furthermore, although it was not statistical significance, there was a dose–response relationship between the number of CHIP mutations and its impact on OS (number of mutations = 1, *β* = 1.56, *p* = 0.005; number of mutations = 2, *β* = 1.75, *p* = 0.154; number of mutations = 3, *β* = 3.01, *p* = 0.115) (Additional file 1: Table S5). However, it was now shown between the percent of VAF and its impact on OS (Additional file 1: Table S5).

To identify the clinical impact of CH according to the adjuvant therapy in early-stage NSCLC, stage IIB patients were further analyzed. There were significant differences in age (*p* < 0.001), the number of comorbidities (*p* = 0.028), and tumor size (*p* = 0.001) between the two groups. The details of baseline characteristics of patients with stage IIB are described in Additional file 1: Table S6. In the univariable analysis, age (HR 1.04, 95% CI 1.01–1.06, *p* = 0.010), DLCO < 60% (HR 2.90, 95% CI 1.45–5.81, *p* = 0.003), CH (HR 2.11, 95% CI 1.21–3.69, *p* = 0.009), and an interaction term between CH and adjuvant therapy (HR 2.42, 95% CI 1.54–3.81, *p* < 0.001) were shown as significant prognostic factor for OS. In addition, the interaction term between CH and adjuvant therapy remained significant in the multivariable Cox analysis (HR 1.19, 95% CI 1.00–1.1.41, *p* = 0.041) (Table 3).

**Table 3:** Univariable and multivariable analysis for overall survival in patients with stage IIB NSCLC

	Univariable analysis		Multivariable analysis	
	HR* (95% CI)	*p*-value	HR (95% CI)	*p*-value
**Presence of CH (VAF ≥ 2.0%)**	2.11 (1.21–3.69)	0.009*	1.71 (0.93–3.14)	0.057
**Adjuvant therapy**				
Done vs. not done	1.04 (0.65–1.70)	0.853		
Presence of CH* adjuvant therapy	2.42 (1.54–3.81)	< 0.001*	1.19 (1.00–1.41)	0.041*
**Age (years)**	1.04 (1.01–1.06)	0.010*	1.03 (1.00–1.06)	0.048*
**History of smoking**	1.30 (0.82–2.03)	0.244		
**Sex (male)**	1.30 (0.82–2.05)	0.259		
**The number of comorbidities**				
1 vs. 0	1.01 (0.64–1.60)	0.960		
≥ 2 vs. 0	0.89 (0.46–1.72)	0.730		
**Pulmonary function**				
FEV1 < 60%	0.53 (0.13–2.14)	0.370		
DLCO < 60%	2.90 (1.45–5.81)	0.003*	1.02 (1.37–5.65)	0.005*
**EGFR mutation**				
No vs. yes	1.03 (0.62–1.72)	0.913		
Unchecked vs. yes	1.32 (0.79–2.22)	0.294		
**Histologic structure**				
SqCC vs. ADC	1.09 (0.69–1.72)	0.703		
Others vs. ADC	0.82 (0.33–2.06)	0.674		

Univariable and multivariable cox regression a﻿nalysis were used for comparison

*NSCLC* Non-small cell lung cancer, *OS* Overall survival, *HR* Hazard ratio, *CI* Confidence interval, *CH* Clonal hematopoiesis, *VAF* Variant allele fraction, *ADC* Adenocarcinoma, *SqCC* Squamous cell carcinoma, *CH* Clonal hematopoiesis

^*^ denotes *p* < 0.05

## Discussion

In this study, we examined the prevalence and the traits for CH in patients with advanced NSCLC. Furthermore, the clinical impact of preoperatively existing CH on survival outcomes was evaluated in overall patients and after PSM. The presence of CH before surgery was significantly associated with an increase in overall mortality, especially in non–lung cancer mortality and mortality of unknown origin. The prognostic effect of CH was the same after adopting a rigorous risk-adjustment methodology to properly adjust the baseline covariates between the two groups.

Most of cytotoxic chemotherapeutics including platinum-based compounds such as cisplatin target DNA replication machinery. As conventional chemotherapies are designed to kill rapidly dividing cells, they cause critical DNA damage resulting in subsequent cell death [21, 22]. However, mutations in DDR genes related to cancer such as TP53, PPM1D, and CHEK2 impairs cell death process which should be normally activated upon DNA damage, leading to a hematopoietic stem cell survival advantage in the setting of cytotoxic drugs [23, 24]. A recent study reported that cancer-related therapies influence evolutionary trajectories of emerging CH clones [25]. Not only DNA repair gene but also genes related to epigenetics and cell survivals those contribute to the clonal expansion advantage are exaggerated by cytotoxic chemotherapy and radiotherapy [26]. From this perspective, we hypothesized that preoperatively existing CH amplifies the series of processes that trigger CH-related adverse outcomes through cancer-related treatments, resulting in poor survival results.

According to our survival analysis, the presence of CH was significantly associated with poor OS (*p* < 0.001) (Fig. 2A). Given the positive correlation between CH and age, we conducted two types of statistical adjustment. After the adjustment of age, sex, smoking history, and the number of comorbidities with multivariable analysis, the prognostic effect of CH was still significant (HR 1.47, 95% CI 1.00–2.16, *p* = 0.046) (Additional file 1: Table S5). In addition, after PSM, all clinical variables, including age, became similar regardless of CH, and patients with CH still had poor OS compared to those without CH (*p* = 0.029) (Fig. 2C). Therefore, we can conclude that the presence of CH is an independent prognostic factor for OS in patients with adjuvant therapy for advanced stage NSCLC.

In terms of the cause of death, we found that the significant difference according to the presence of CH was shown not in lung cancer mortality (*p* = 0.568), but in non–lung cancer mortality (*p* = 0.042), and mortality of unknown origin (*p* = 0.018). Judging from the good compliance to postoperative surveillance in the patients who completed the adjuvant therapy, it is speculated that deaths from unknown origin were due to acute events, such as cardiopulmonary disease, sepsis, or stroke, rather than a relatively slow progression of cancer. In this analysis, PPM1D gene emerged as a particularly adverse prognostic factor. While PPM1D is commonly known to have mutations primarily induced after chemotherapy [23], mutations can also arise from smoking and various chemical hazards which are associated with the onset of NSCLC [27]. Therefore, we surmise that patients with pre-existing PPM1D mutations before chemotherapy might experience an exacerbated selective amplification of PPM1D following chemotherapy. This intensified amplification might further deteriorate the prognosis, potentially leading to known complications such as worsening heart failure [28] and changes in the immune system [29]. Thus, we believe that these findings support our hypothesis that various adverse outcomes related to CH are amplified by CTx or RTx in patients with CH, which in turn affects survival.

An important outstanding question is how should physicians manage lung cancer patients with CH mutations who are indicated for adjuvant therapy? First, patients who have a high risk of developing adverse outcomes should be distinguished from those who do not. Although there is no clear definition for high-risk CH, the presence of significant blood count abnormalities, a single CH mutation at a high VAF (> 10%), multiple CH mutations, variants in TP53 and PPM1D, DNMT3A variants, and hotspot mutations of IDH1/2 are considered to put patients in the high-risk group [2, 30]. Second, it should be proceeded to refine the patient group where adjuvant therapy is beneficial to prognosis even at the risk of survival loss due to CH. To answer this question, we focused on early-stage NSCLC, i.e., stage II. Because absolute benefit of adjuvant chemotherapy in early-stage NSCLC is 4% in 5 years with cytotoxic backbone [31], we hypothesized that presence of CH might abolish the oncologic benefit of adjuvant chemotherapy in early-stage NSCLC via non-cancer related mortality. Because adjuvant chemotherapy is not prevalently performed in stage I NSCLC in Korea due to reimbursement policy, we focused on stage II disease.

In the univariable analysis of OS for patients with stage IIB NSCLC, adjuvant therapy had a significant interaction with the presence of CH (HR 2.42, 95% CI 1.54–1.3.81, *p* < 0.001). The interaction between adjuvant therapy and CH remained significant in the multivariable analysis (HR 1.19, 95% CI 1.00–1.1.41, *p* = 0.041). It means preoperative CH mutations might amplify the negative prognostic impact on OS when patients with stage IIB NSCLC receive adjuvant therapy. In addition to conventional systemic therapy, the prognostic role of CH is getting attention in patients who underwent immunotherapy that target inhibitory immune cell checkpoints such as PD-1. According to the recent study, CH was associated with worse OS when patients with solid cancers, including non-small cell lung cancer, received immunotherapy [32]. Therefore, given the absolute survival benefit of adjuvant therapy and the potential impact of CH on prognosis, adjuvant therapy should be determined more carefully in patients with preoperatively existing CH mutations through a multidisciplinary approach.

This study had notable limitations. First, selection bias is inherent in a retrospective study from a single institution; however, as the data in this study were gathered prospectively, we aimed to minimize this bias as much as possible. Second, the number of patients enrolled in this study was relatively small, which may raise the possibility of selection bias. Indeed, some findings, which might have seemed different, were not significant. However, high-depth (1000X) sequencing data used in our study enables identification of CH status in detail unlike public cohort including UK Biobank and the Cancer Genome Atlas. Third, the outcomes in this study were not validated in an independent cohort, which remains the questions about reproducibility. Thus, it is required to perform an external validation with an independent data set containing sufficient sample size. Finally, some patients died of unknown origin, which limited the accurate assessment of CH-related adverse outcomes. In addition, we would like to mention that low sequencing depth of public data including the Cancer Genome Atlas was not suitable for validation of our detailed clinical findings due to insufficient capture of patients harboring CH.

## Conclusions

In resected NSCLC, preoperatively existing CH mutations have a significant clinical impact on patients with NSCLC who received surgery followed by adjuvant therapy, which decreases the survival outcome. Especially, this phenomenon is clearly demonstrated in early-stage NSCLC. Research efforts to validate our results are encouraged and will help to reestablish our approach to managing CH in adjuvant therapy settings for NSCLC.

### Supplementary Information

**Additional file 1.**

**Additional file 2.**

## Data Availability

All data supporting the findings of this study are available within the paper and its supplementary information. Microsatellite primer sequences are provided in Supplementary Table 2, along with original reference describing the microsatellites used in this study.

## Abbreviations

CH: Clonal hematopoiesis
CHIP: Clonal hematopoiesis of indeterminate potential
CRTx: Chemoradiation therapy
CTx: Chemotherapy
DDR: DNA damage response
HSCs: Hematopoietic stem cells
HR: Hazard ratio
NGS: Next-generation sequencing
NSCLC: Non-small cell lung cancer
OS: Overall survival
PSM: Propensity score matching
RFS: Recurrence-free survival
RTx: Radiotherapy
SMDs: Standardized mean differences
VAF: Variant allele frequency
