# Supplementary material for: Pre-operative clonal hematopoiesis is related to adverse outcome in lung cancer after adjuvant therapy
Source: Genome Med. 2023 Dec 12;15:111. doi: 10.1186/s13073-023-01266-4 (PMC10714617; doi:10.1186/s13073-023-01266-4)

Table S1. Eighty-nine CH genes targeted in the NGS panel and reference source.

| APC | ASXL1 | ASXL2 | ATM | BCL11B | BCOR | BCORL1 | BIRC3 |
| --- | --- | --- | --- | --- | --- | --- | --- |
| BRAF | BRCC3 | CARD11 | CASP8 | CBL | CD58 | CD79B | CNOT3 |
| CREBBP | CUX1 | DDX3X | DNMT3A | EP300 | ETV6 | EZH2 | FAM46C |
| FBXW7 | FLT3 | FOXP1 | GNAS | GNB1 | GPS2 | HIST1H1C | IDH2 |
| IKZF1 | IKZF2 | JAK1 | JAK2 | JAK3 | JARID2 | KDM6A | KIT |
| KLHL6 | KRAS | LUC7L2 | MAP3K1 | KMT2D | MPL | MYD88 | NF1 |
| NFE2L2 | NOTCH1 | NOTCH2 | NRAS | PDS5B | PDSS2 | PHF6 | PHIP |
| PIK3CA | PIK3R1 | PPM1D | PRDM1 | PRPF40B | PTEN | PTPN11 | RAD21 |
| RIT1 | RPS15 | SETD2 | SETDB1 | SF1 | SF3A1 | SF3B1 | SMC1A |
| SMC3 | SRSF2 | STAG1 | STAG2 | STAT3 | SUZ12 | TBL1XR1 | TET1 |
| TET2 | TNFAIP3 | TNFRSF14 | TP53 | U2AF1 | VHL | WT1 | ZRSR2 |
| CHEK2 |  |  |  |  |  |  |  |

| **Cohort types** | **Papers** | **DOI** |
| --- | --- | --- |
| Cancer survivors | 2018 CCR, Coombs et al. | 10.1158/1078-0432.CCR-18-1201 |
| Cancer survivors | 2018 JAMA, Ptashkin et al. | 10.1001/jamaoncol.2018.2297 |
| Cancer survivors | 2017 CSC, Coombs et al. | 10.1016/j.stem.2017.07.010 |
| TMN | 2017 Lancet, Kevin et al. | 10.1016/S1470-2045(16)30626-X |
| TMN | 2017 Lancet, Gillis et al. | 10.1016/S1470-2045(16)30627-1 |
| Healthy | 2019 HereditaryCancer, Slavin et al. | 10.1016/j.cancergen.2019.04.005 |
| Healthy | 2017 NEJM, Jaiswal et al. | 10.1056/NEJMoa1701719 |
| Healthy | 2014 NEJM, Jaiswal et al. | 10.1056/NEJMoa1408617 |
| Healthy | 2014 NEJM, Genovese et al. | 10.1056/NEJMoa1409405 |

Table S2. List of detected CH mutation in the study cohort

| **Gene** | **Chromosome** | **Position** | **Variant classification** | **Reference allele** | **Alternate allele** | **Protein change** | **CDS** | **VAF** | **PD** |
| --- | --- | --- | --- | --- | --- | --- | --- | --- | --- |
| ASXL1 | chr20 | 31022441 | FRAME_SHIFT | A | AG | p.642Gly_643Glyfs | c.1927_1928insG | 3.3 | 1 |
| ASXL1 | chr20 | 31022286 | STOP_GAINED | T | TA | p.590Tyr_591GlninsTer??? | c.1772_1773insA | 2.2 | 1 |
| ASXL1 | chr20 | 31021211 | STOP_GAINED | C | T | p.Arg404* | c.1210C>T | 30 | 1 |
| ASXL1 | chr20 | 31022441 | FRAME_SHIFT | A | AG | p.642Gly_643Glyfs | c.1927_1928insG | 8 | 1 |
| ASXL1 | chr20 | 31023478 | FRAME_SHIFT | ACTCTGAAGCACTGAGTC | A | p.987Asp_994Hisfs | c.2964_2971delCTCTGAAGCACTGAGTC | 9.6 | 1 |
| ASXL1 | chr20 | 31024343 | FRAME_SHIFT | TC | T | p.1276Pro_1277Asnfs | c.3829_3830delC | 2.3 | 1 |
| ASXL1 | chr20 | 31022402 | FRAME_SHIFT | TCACCACTGCCATAGAGAGGCGGC | T | p.629His_637Thrfs | c.1888_1896delCACCACTGCCATAGAGAGGCGGC | 16.4 | 1 |
| ASXL1 | chr20 | 31022927 | FRAME_SHIFT | A | AC | p.804Pro_805Thrfs | c.2413_2414insC | 10.6 | 1 |
| ASXL1 | chr20 | 31022700 | FRAME_SHIFT | AG | A | p.728Ser_729Cysfs | c.2186_2187delG | 5.8 | 1 |
| ASXL1 | chr20 | 31022463 | FRAME_SHIFT | GGT | G | p.649Gly_650Glyfs | c.1949_1950delGT | 9.2 | 1 |
| ASXL1 | chr20 | 31022441 | FRAME_SHIFT | A | AG | p.642Gly_643Glyfs | c.1927_1928insG | 3.1 | 1 |
| ASXL1 | chr20 | 31022288 | STOP_GAINED | C | A | p.Tyr591* | c.1773C>A | 2.4 | 1 |
| ASXL1 | chr20 | 31022936 | FRAME_SHIFT | TC | T | p.807Pro_808Alafs | c.2422_2423delC | 24.6 | 1 |
| ASXL1 | chr20 | 31022441 | FRAME_SHIFT | A | AG | p.642Gly_643Glyfs | c.1927_1928insG | 11.7 | 1 |
| ATM | chr11 | 108143318 | NON_SYNONYMOUS_CODING | T | A | p.Leu1046His | c.3137T>A | 23.2 | 0 |
| ATM | chr11 | 108206595 | NON_SYNONYMOUS_CODING | T | A | p.Asp2725Glu | c.8175T>A | 7.3 | 0 |
| ATM | chr11 | 108099994 | NON_SYNONYMOUS_CODING | A | C | p.Lys92Thr | c.275A>C | 28.8 | 0 |
| BCL11B | chr14 | 99642343 | NON_SYNONYMOUS_CODING | G | T | p.Ser277Tyr | c.830C>A | 3.3 | 0 |
| CARD11 | chr7 | 2974263 | NON_SYNONYMOUS_CODING | T | C | p.Ser448Gly | c.1342A>G | 5 | 0 |
| CARD11 | chr7 | 2956964 | NON_SYNONYMOUS_CODING | C | T | p.Arg888His | c.2663G>A | 4.8 | 0 |
| CBL | chr11 | 119148973 | NON_SYNONYMOUS_CODING | A | G | p.His398Arg | c.1193A>G | 2.6 | 1 |
| CD79B | chr17 | 62008706 | NON_SYNONYMOUS_CODING | C | T | p.Arg37Gln | c.110G>A | 3.5 | 0 |
| CHEK2 | chr22 | 29090079 | NON_SYNONYMOUS_CODING | C | T | p.Val511Ile | c.1531G>A | 2.1 | 0 |
| CHEK2 | chr22 | 29083949 | FRAME_SHIFT | CG | C | p.565Arg_566Glufs | c.1696_1697delC | 2.9 | 1 |
| CUX1 | chr7 | 101459346 | STOP_GAINED | G | A | p.Trp12* | c.36G>A | 3.2 | 1 |
| DNMT3A | chr2 | 25463600 | SPLICE_SITE_ACCEPTOR | C | T | - | - | 2.9 | 1 |
| DNMT3A | chr2 | 25462077 | NON_SYNONYMOUS_CODING | G | C | p.Pro777Arg | c.2330C>G | 2.9 | 1 |
| DNMT3A | chr2 | 25464456 | NON_SYNONYMOUS_CODING | T | A | p.Asp686Val | c.2057A>T | 20.3 | 0 |
| DNMT3A | chr2 | 25468888 | SPLICE_SITE_DONOR | C | T | - | - | 25.1 | 1 |
| DNMT3A | chr2 | 25468201 | SPLICE_SITE_ACCEPTOR | TC | T | - | - | 13 | 1 |
| DNMT3A | chr2 | 25462018 | NON_SYNONYMOUS_CODING | T | C | p.Asn797Asp | c.2389A>G | 2 | 1 |
| DNMT3A | chr2 | 25463286 | NON_SYNONYMOUS_CODING | C | T | p.Arg736His | c.2207G>A | 12.9 | 1 |
| DNMT3A | chr2 | 25467168 | FRAME_SHIFT | CG | C | p.568Pro_569Glyfs | c.1706_1707delC | 3 | 1 |
| DNMT3A | chr2 | 25469079 | FRAME_SHIFT | C | CAT | p.459Ser_460Thrfs | c.1378_1379insTA | 8.5 | 1 |
| DNMT3A | chr2 | 25469986 | FRAME_SHIFT | AC | A | p.351Ser_352Alafs | c.1055_1056delG | 4.5 | 1 |
| DNMT3A | chr2 | 25467449 | NON_SYNONYMOUS_CODING | C | A | p.Gly543Cys | c.1627G>T | 18 | 1 |
| DNMT3A | chr2 | 25467071 | FRAME_SHIFT | GC | G | p.600Trp_601Profs | c.1803_1804delG | 8.9 | 1 |
| DNMT3A | chr2 | 25466800 | NON_SYNONYMOUS_CODING | G | C | p.Arg635Gly | c.1903C>G | 9.6 | 0 |
| DNMT3A | chr2 | 25464433 | NON_SYNONYMOUS_CODING | G | A | p.His694Tyr | c.2080C>T | 4.4 | 0 |
| DNMT3A | chr2 | 25469504 | FRAME_SHIFT | GGCCCTTAGGGCCA | G | p.416Ser_421Leufs | c.1251_1256delCGGGAATCCCGGT | 2 | 1 |
| DNMT3A | chr2 | 25457243 | NON_SYNONYMOUS_CODING | G | A | p.Arg882Cys | c.2644C>T | 2.3 | 1 |
| DNMT3A | chr2 | 25469527 | FRAME_SHIFT | AAGCCCCCC | A | p.410Leu_414Glnfs | c.1233_1237delTCGGGGGG | 2 | 1 |
| DNMT3A | chr2 | 25462068 | NON_SYNONYMOUS_CODING | A | G | p.Ile780Thr | c.2339T>C | 2.5 | 1 |
| DNMT3A | chr2 | 25457243 | NON_SYNONYMOUS_CODING | G | A | p.Arg882Cys | c.2644C>T | 3.4 | 1 |
| DNMT3A | chr2 | 25458595 | FRAME_SHIFT | A | AT | p.858Leu_859Trpfs | c.2577_2578insA | 12.1 | 1 |
| DNMT3A | chr2 | 25469531 | NON_SYNONYMOUS_CODING | C | T | p.Gly413Ser | c.1237G>A | 2 | 0 |
| DNMT3A | chr2 | 25470028 | SPLICE_SITE_ACCEPTOR | C | T | - | - | 6.4 | 1 |
| DNMT3A | chr2 | 25467470 | FRAME_SHIFT | AG | A | p.534Ser_535Tyrfs | c.1605_1606delC | 8.9 | 1 |
| DNMT3A | chr2 | 25457243 | NON_SYNONYMOUS_CODING | G | A | p.Arg882Cys | c.2644C>T | 2.9 | 1 |
| DNMT3A | chr2 | 25462047 | NON_SYNONYMOUS_CODING | G | A | p.Ala787Val | c.2360C>T | 3.5 | 0 |
| DNMT3A | chr2 | 25458595 | NON_SYNONYMOUS_CODING | A | G | p.Trp860Arg | c.2578T>C | 2.2 | 1 |
| DNMT3A | chr2 | 25463172 | NON_SYNONYMOUS_CODING | T | A | p.Glu774Val | c.2321A>T | 2.7 | 1 |
| DNMT3A | chr2 | 25467208 | SPLICE_SITE_ACCEPTOR | C | G | - | - | 9.6 | 1 |
| DNMT3A | chr2 | 25470472 | FRAME_SHIFT | GC | G | p.333Gly_334Lysfs | c.1001_1002delG | 2.4 | 1 |
| DNMT3A | chr2 | 25469633 | NON_SYNONYMOUS_CODING | G | A | p.Arg379Cys | c.1135C>T | 9.1 | 0 |
| DNMT3A | chr2 | 25467190 | NON_SYNONYMOUS_CODING | C | T | p.Cys562Tyr | c.1685G>A | 7.2 | 1 |
| DNMT3A | chr2 | 25470497 | NON_SYNONYMOUS_CODING | C | A | p.Arg326Leu | c.977G>T | 28.5 | 0 |
| DNMT3A | chr2 | 25467023 | SPLICE_SITE_DONOR | C | T | - | - | 2 | 1 |
| DNMT3A | chr2 | 25457159 | FRAME_SHIFT | C | CAA | p.908Phe_909Alafs | c.2727_2728insTT | 3.7 | 1 |
| DNMT3A | chr2 | 25458591 | NON_SYNONYMOUS_CODING | C | T | p.Cys861Tyr | c.2582G>A | 4.2 | 0 |
| ETV6 | chr12 | 11992181 | NON_SYNONYMOUS_CODING | G | A | p.Gly91Ser | c.271G>A | 19.3 | 0 |
| FOXP1 | chr3 | 71026147 | NON_SYNONYMOUS_CODING | T | C | p.Tyr492Cys | c.1475A>G | 2.8 | 1 |
| FOXP1 | chr3 | 71064710 | FRAME_SHIFT | A | ATT | p.320Gln_321Serfs | c.963_964insAA | 3.8 | 1 |
| JAK2 | chr9 | 5073770 | NON_SYNONYMOUS_CODING | G | T | p.Val617Phe | c.1849G>T | 3.1 | 1 |
| KMT2D | chr12 | 49435269 | NON_SYNONYMOUS_CODING | C | T | p.Arg2095His | c.6284G>A | 3.1 | 0 |
| KMT2D | chr12 | 49434156 | STOP_GAINED | A | T | p.Leu2466* | c.7397T>A | 2.3 | 1 |
| MAP3K1 | chr5 | 56177549 | NON_SYNONYMOUS_CODING | T | C | p.Leu841Pro | c.2522T>C | 4.4 | 0 |
| MPL | chr1 | 43815009 | NON_SYNONYMOUS_CODING | G | T | p.Trp515Leu | c.1544G>T | 3.1 | 1 |
| NF1 | chr17 | 29552132 | NON_SYNONYMOUS_CODING | G | A | p.Cys622Tyr | c.1865G>A | 3.1 | 0 |
| NOTCH1 | chr9 | 139390743 | NON_SYNONYMOUS_CODING | G | A | p.Thr2483Met | c.7448C>T | 4.2 | 1 |
| NOTCH2 | chr1 | 120612014 | NON_SYNONYMOUS_CODING | C | T | p.Ala3Thr | c.7G>A | 25.3 | 0 |
| PHIP | chr6 | 79787592 | NON_SYNONYMOUS_CODING | G | A | p.Pro27Ser | c.79C>T | 7.6 | 0 |
| PPM1D | chr17 | 58734012 | NON_SYNONYMOUS_CODING | G | A | p.Arg357His | c.1070G>A | 24.1 | 0 |
| PPM1D | chr17 | 58740438 | FRAME_SHIFT | AT | A | p.447Asn_448Phefs | c.1344_1345delT | 3.4 | 1 |
| PPM1D | chr17 | 58740357 | FRAME_SHIFT | C | CA | p.420Ser_421Leufs | c.1263_1264insA | 5.1 | 1 |
| PPM1D | chr17 | 58740532 | FRAME_SHIFT | T | TA | p.479Lys_480Alafs | c.1438_1439insA | 6.6 | 1 |
| PPM1D | chr17 | 58740505 | FRAME_SHIFT | T | TC | p.470Pro_471Glufs | c.1411_1412insC | 6.9 | 1 |
| PPM1D | chr17 | 58740749 | STOP_GAINED | C | T | p.Arg552* | c.1654C>T | 13.9 | 1 |
| PPM1D | chr17 | 58740623 | FRAME_SHIFT | C | CA | p.509Gln_510Lysfs | c.1529_1530insA | 3.5 | 1 |
| PPM1D | chr17 | 58740694 | FRAME_SHIFT | CT | C | p.533Phe_534Lysfs | c.1600_1601delT | 2.9 | 1 |
| PRPF40B | chr12 | 50027317 | FRAME_SHIFT | G | GGATGACCTAGAGGGTGA | p.189Asp_190Aspfs | c.568_569insGATGACCTAGAGGGTGA | 18.3 | 1 |
| PRPF40B | chr12 | 50027317 | FRAME_SHIFT | G | GGATGACCTAGAGGGTGA | p.189Asp_190Aspfs | c.568_569insGATGACCTAGAGGGTGA | 18.3 | 1 |
| RAD21 | chr8 | 117875498 | NON_SYNONYMOUS_CODING | C | T | p.Val49Met | c.145G>A | 2.6 | 0 |
| SETD2 | chr3 | 47139554 | NON_SYNONYMOUS_CODING | C | T | p.Cys1678Tyr | c.5033G>A | 2 | 0 |
| SF3B1 | chr2 | 198260972 | NON_SYNONYMOUS_CODING | A | G | p.Ile1116Thr | c.3347T>C | 3.3 | 0 |
| SF3B1 | chr2 | 198267359 | NON_SYNONYMOUS_CODING | C | A | p.Lys666Asn | c.1998G>T | 4.1 | 1 |
| SF3B1 | chr2 | 198267360 | NON_SYNONYMOUS_CODING | T | C | p.Lys666Arg | c.1997A>G | 20.5 | 1 |
| TET1 | chr10 | 70406293 | NON_SYNONYMOUS_CODING | A | C | p.Lys1269Asn | c.3807A>C | 24.4 | 0 |
| TET2 | chr4 | 106157878 | FRAME_SHIFT | GT | G | p.926Val_927Phefs | c.2780_2781delT | 2.5 | 1 |
| TET2 | chr4 | 106182979 | FRAME_SHIFT | CT | C | p.1339Leu_1340Alafs | c.4019_4020delT | 2.6 | 1 |
| TET2 | chr4 | 106193794 | FRAME_SHIFT | CTT | C | p.1418Pro_1420Tyrfs | c.4257_4259delTT | 3.2 | 1 |
| TET2 | chr4 | 106196243 | STOP_GAINED | C | T | p.Gln1526* | c.4576C>T | 4.6 | 1 |
| TET2 | chr4 | 106155853 | FRAME_SHIFT | A | ATT | p.251Ile_252Asnfs | c.755_756insTT | 5.2 | 1 |
| TET2 | chr4 | 106180891 | NON_SYNONYMOUS_CODING | A | G | p.Arg1307Gly | c.3919A>G | 2.8 | 0 |
| TET2 | chr4 | 106180853 | NON_SYNONYMOUS_CODING | A | G | p.Tyr1294Cys | c.3881A>G | 20.3 | 1 |
| TET2 | chr4 | 106157989 | FRAME_SHIFT | C | CA | p.963Gln_964Thrfs | c.2891_2892insA | 17.6 | 1 |
| TET2 | chr4 | 106157969 | FRAME_SHIFT | T | TA | p.956Leu_957Glnfs | c.2871_2872insA | 2.1 | 1 |
| TET2 | chr4 | 106155281 | FRAME_SHIFT | A | AT | p.60Tyr_61Glyfs | c.183_184insT | 2 | 1 |
| TET2 | chr4 | 106157789 | FRAME_SHIFT | AG | A | p.896Gln_897Glyfs | c.2691_2692delG | 14.3 | 1 |
| TET2 | chr4 | 106157560 | STOP_GAINED | C | T | p.Gln821* | c.2461C>T | 4.5 | 1 |
| TNFAIP3 | chr6 | 138197265 | FRAME_SHIFT | A | AT | p.255His_256Phefs | c.768_769insT | 2.6 | 1 |
| TNFAIP3 | chr6 | 138196885 | STOP_GAINED | C | T | p.Arg183* | c.547C>T | 3.1 | 1 |
| TNFAIP3 | chr6 | 138200309 | FRAME_SHIFT | CGCATTCTTGCCACA | C | p.575Pro_581Alafs | c.1728_1734delGCATTCTTGCCACA | 6.3 | 1 |
| TP53 | chr17 | 7574003 | STOP_GAINED | G | A | p.Arg342* | c.1024C>T | 28.1 | 1 |
| U2AF1 | chr21 | 44524456 | NON_SYNONYMOUS_CODING | G | T | p.Ser34Tyr | c.101C>A | 8.8 | 1 |
| ZRSR2 | chrX | 15833956 | FRAME_SHIFT | CT | C | p.238Phe_239Tyrfs | c.715_716delT | 3.3 | 1 |

Table S3. Baseline characteristics of patients who underwent adjuvant therapy for stage IIB or III according to the presence of clonal hematopoiesis after propensity score matching (final cohort).

| Variables | CH (+)  (n=86) | CH (-)  (n=172) | *p* value | ASMD |
| --- | --- | --- | --- | --- |
| **Age (year)** | 63.0 ± 8.2 | 62.4 ± 8.6 | 0.620 | 0.066 |
| **Sex (male)** | 60 (69.8) | 115 (66.9) | 0.742 | 0.063 |
| **History of smoking** | 53 (61.6) | 105 (61.0) | 1.000 | 0.012 |
| **The number of comorbidities** |  |  | 0.278 | 0.183 |
| 0 | 46 (53.5) | 74 (43.0) |  |  |
| 1 | 26 (30.2) | 62 (36.0) |  |  |
| ≥ 2 | 14 (16.3) | 36 (20.9) |  |  |
| **Pulmonary function** |  |  |  |  |
| FEV1 < 60% | 4 (4.7) | 5 (2.9) | 0.719 | 0.092 |
| DLCO < 60% | 6 (7.0) | 9 (5.2) | 0.778 | 0.073 |
| **Histologic structure** |  |  | 0.777 | 0.093 |
| ADC* | 54 (62.8) | 114 (66.3) |  |  |
| SqCC* | 24 (27.9) | 41 (23.8) |  |  |
| Others | 8 (9.3) | 17 (9.9) |  |  |
| **Maximal tumor size (mm)** | 40.6 ± 17.3 | 39.7 ± 16.5 | 0.867 | 0.022 |
| **EGFR mutation** |  |  | 0.839 | 0.131 |
| Yes | 22 (25.6) | 14 (22.6) |  |  |
| No | 22 (25.6) | 20 (32.3) |  |  |
| Unchecked | 42 (48.8) | 28 (45.2) |  |  |
| **Pathological T factor** |  |  | 0.425 | 0.174 |
| T1 | 22 (25.6) | 34 (19.8) |  |  |
| T2 | 32 (37.2) | 82 (47.7) |  |  |
| T3 | 25 (29.1) | 42 (24.4) |  |  |
| T4 | 7 (8.1) | 14 (8.1) |  |  |
| **Pathological N factor** |  |  | 0.817 | 0.084 |
| N0 | 9 (10.5) | 19 (11.0) |  |  |
| N1 | 35 (40.7) | 63 (36.6) |  |  |
| N2 | 42 (48.8) | 90 (52.3) |  |  |
| **Pathological stage** |  |  | 0.830 | 0.080 |
| IIB | 31 (36.0) | 61 (35.5) |  |  |
| IIIA | 44 (51.2) | 93 (54.1) |  |  |
| IIIB | 11 (12.8) | 18 (10.5) |  |  |
| **Type of adjuvant therapy** |  |  | 0.965 | 0.023 |
| CTx | 45 (52.3) | 92 (53.5) |  |  |
| CRTx | 41 (47.7) | 80 (46.5) |  |  |

Data are presented as no. (%) unless noted otherwise. CH: Clonal hematopoiesis, FEV1: Forced expiratory volume during the first second, DLCO: Diffusing capacity for carbon monoxide, ADC: Adenocarcinoma, SqCC: Squamous cell carcinoma, EGFR: Epidermal growth factor receptor, CRTx: Chemoradiotherapy, CTx: Chemotherapy.

Table S4. Cause of deaths for the whole period of observation

| Variables | Total  (=415) | CH (+)  (n=86) | CH (-)  (n=329) | p value |
| --- | --- | --- | --- | --- |
| Lung cancer mortality | 106 (25.5%) | 23 (26.7%) | 83 (25.2%) | 0.882 |
| Non-lung cancer related | 33 (8.0%) | 11 (12.8%) | 22 (6.7%) | 0.101 |
| Cardiopulmonary | 23 (5.3%) | 8 (9.3%) | 14 (4.3%) | 0.112 |
| Secondary malignancy | 3 (0.7%) | 0 | 3 (0.9%) | 0.862 |
| Infection | 4 (1.0%) | 1 (1.2%) | 3 (0.9%) | 1.000 |
| Stroke | 4 (1.0%) | 2 (2.3%) | 2 (0.6%) | 0.191 |
| Unknown origin | 36 (8.7%) | 12 (14.0%) | 24 (7.3%) | 0.082 |

Table S5. Sensitivity analysis of the association between clonal hematopoiesis and overall survival.

|  | Number |  |  |  | Overall survival | | | | |
| --- | --- | --- | --- | --- | --- | --- | --- | --- | --- |
|  |  |  | Univariable analysis | | | |  | Multivariable analysis* | |
|  | (+) vs. (-) |  | HR* (95% CI) | | | P value |  | HR (95% CI) | P value |
| By genes (VAF ≥ 2.0%) |  |  |  | | |  |  |  |  |
| All CH genes | 86 vs. 329 |  | 1.56 (1.18–2.47) | | | 0.005 |  | 1.47 (1.00–2.16) | 0.046 |
| DNMT3A | 34 vs. 381 |  | 1.33 (0.76–2.31) | | | 0.315 |  | 1.31 (0.75–2.28) | 0.348 |
| Non-DNMT3A | 52 vs. 363 |  | 1.59 (1.02–2.49) | | | 0.042 |  | 1.45 (0.91–2.30) | 0.110 |
| TET2 | 11 vs. 404 |  | 1.03 (0.38–2.78) | | | 0.956 |  | 0.89 (0.33–2.43) | 0.825 |
| ASXL1 | 14 vs. 401 |  | 1.16 (0.48–2.84) | | | 0.745 |  | 1.14 (0.46–2.80) | 0.783 |
| PPM1D | 7 vs. 408 |  | 4.35 (1.91–9.89) | | | <0.001 |  | 4.93 (2.13–11.40) | <0.001 |
| DDR* | 13 vs. 402 |  | 2.12 (1.00–4.56) | | | 0.045 |  | 2.32 (1.08–4.98) | 0.031 |
| By mutation number (VAF ≥ 2.0%) | |  |  | | |  |  |  |  |
| ≥ 1 vs. 0 | 86 vs. 329 |  | 1.56 (1.18–2.47) | | | 0.005 |  | 1.47 (1.00–2.16) | 0.046 |
| ≥ 2 vs. 0 | 15 vs. 329 |  | 1.75 (0.81–3.76) | | | 0.154 |  | 1.73 (0.80–3.76) | 0.163 |
| ≥ 3 vs. 0 | 3 vs. 329 |  | 3.01 (0.76–12.57) | | | 0.115 |  | 3.58 (0.86–14.82) | 0.079 |
| By % of VAF | |  |  | | |  |  |  |  |
| ≥ 2.0% vs. < 2.0% | 86 vs. 329 |  | 1.56 (1.18–2.47) | | | 0.005 |  | 1.47 (1.00–2.16) | 0.046 |
| ≥ 5.0% vs. < 2.0% | 38 vs. 329 |  | 1.47 (0.97–2.48) | | | 0.157 |  | 1.37 (0.80–2.34) | 0.245 |
| ≥ 10.0% vs. < 2.0% | 20 vs. 329 |  | 1.10 (0.51–2.38) | | | 0.804 |  | 0.99 (0.46–2.14) | 0.976 |

CH, clonal hematopoiesis; HR, hazard ratio; CI, confidence interval; VAF, variant allele fraction; DDR, DNA damage response.

* CH was adjusted by age, sex, smoking history, and the number of comorbidities in the multivariable analysis.

* DDR clones include PPM1D, TP53, and CHEK2.

Table S6. Baseline characteristics of patients with stage IIB NSCLC according to the performance of adjuvant therapy.

| Variables | Adjuvant therapy | | | P value |  |
| --- | --- | --- | --- | --- | --- |
|  | Total  (n=289) | Done  (n=170) | Not done  (n=119) |  |  |
| **Age (year)** | 62.4±8.4 | 59.6±7.6 | 66.4±7.9 | <0.001 |  |
| **Sex (male)** | 192 (66.4) | 118 (69.4) | 74 (62.2) | 0.249 |  |
| **History of smoking** | 189 (61.6) | 104 (61.2) | 74 (62.2) | 0.960 |  |
| **The number of comorbidities** |  |  |  | 0.028 |  |
| 0 | 148 (51.2) | 98 (57.6) | 50 (42.0) |  |  |
| 1 | 101 (34.9) | 53 (31.2) | 48 (40.3) |  |  |
| ≥ 2 | 40 (13.8) | 19 (11.2) | 21 (17.6) |  |  |
| **Pulmonary function** |  |  |  |  |  |
| FEV1 < 60% | 12 (4.2) | 5 (2.9) | 7 (5.9) | 0.350 |  |
| DLCO < 60% | 17 (5.9) | 8 (6.7) | 9 (5.3) | 0.800 |  |
| **Histologic structure** |  |  |  | 0.520 |  |
| ADC* | 174 (60.2) | 102 (60.0) | 72 (60.5) |  |  |
| SqCC* | 96 (33.2) | 59 (34.7) | 37 (31.1) |  |  |
| Others | 19 (6.6) | 9 (5.3) | 10 (8.4) |  |  |
| **Maximal tumor size (mm)** | 38.8±14.6 | 36.3±13.0 | 42.3±15.9 | 0.001 |  |
| **EGFR mutation** |  |  |  | 0.249 |  |
| Yes | 157 (54.3) | 90 (52.9) | 67 (56.3) |  |  |
| No | 70 (24.2) | 38 (22.4) | 32 (26.9) |  |  |
| Unchecked | 62 (21.5) | 42 (24.7) | 20 (16.8) |  |  |

Data presented as no. (%) unless noted otherwise. CH, clonal hematopoiesis; ASMD, absolute standardized mean difference; FEV1, forced expiratory volume during the first second; DLCO, diffusing capacity for carbon monoxide; ADC, adenocarcinoma; SqCC, squamous cell carcinoma; CRTx, chemoradiotherapy; CTx, chemotherapy.


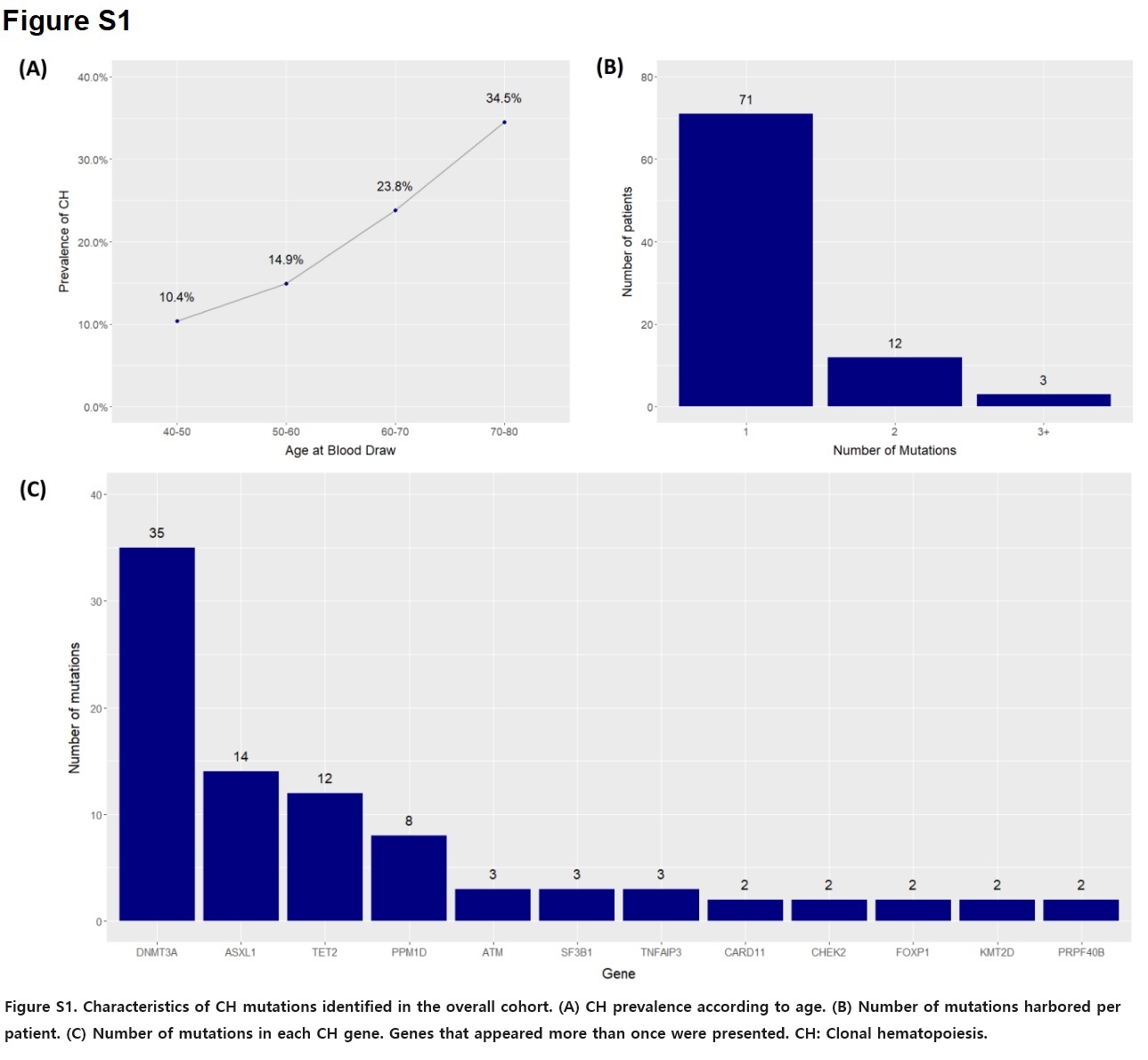

Supplement: Supplementary file 1 — Additional file 1. [file 13073_2023_1266_MOESM1_ESM.docx]
